# Exploring hazard anticipation and stress while driving in light of defensive behavior theory

**DOI:** 10.1038/s41598-023-34714-7

**Published:** 2023-05-15

**Authors:** Laora Kerautret, Stephanie Dabic, Jordan Navarro

**Affiliations:** 1 Valeo Interior Controls, Rue Jules Verne, 74100 Annemasse, France; 2 Laboratoire d’Etude des Mecanismes Cognitifs (EA 3082), University Lyon 2, 5 Avenue Pierre Mendès, 69676 Bron, France; 3 Institut Universitaire de France, Paris, France

**Keywords:** Physiology, Psychology

## Abstract

In driving, poor hazard anticipation would provide drivers less time to prepare an appropriate response, increasing the urgency of the situation and generating more stress. Assuming this, the current study seeks to determine whether a predictable road hazard triggers hazard anticipation in drivers that can mitigate the ensuing stress response, and whether the stress response is influenced by driving experience. In a simulated road environment, a cue was used to trigger hazard anticipation, and a road hazard to induce a stress response. Heart rate, pupil diameter, driving speed, subjective stress, arousal, and negative emotions, were retrieved from 36 drivers who all faced the cue followed by the hazard (i.e. a predictable hazard), the cue only, and the hazard only. In the light of work on defensive behaviors, the findings indicate that a predictable hazard triggers hazard anticipation detectable via (1) freezing behavior—characterized by cardiac deceleration—(2) anticipatory pupil dilation and (3) anticipatory speed deceleration. The results also point to a beneficial role for hazard anticipation in reducing driver stress, as evidenced by reductions in peak heart rate levels, as well as in reported levels of stress and negative emotions. Finally, the findings showed an influence of driving experience on reported levels of stress. Overall, this study shows how previous work on defensive behaviors can be used to gain insight into the processes and driving behaviors involved in hazard anticipation and stress.

## Introduction

During a driving task, drivers frequently encounter situations involving road hazards. The likelihood of encountering hazards is so high that hazard avoidance has been identified as one of the three general subtasks of driving, along with navigation and control^1^. When a hazard is encountered, drivers need to detect it, then understand what makes it hazardous, and finally anticipate the movement or behavior of the hazard^2,3^. This driving skill, also known as hazard perception, is the only one that has been consistently implicated in crashes^4,5^. So far, hazard perception has been extensively studied using tests in which drivers were asked to detect potential hazards in filmed road scenes^6–9^. Although these tests have been shown to be relatively effective, in particular, in predicting collisions^5,10^ and in discriminating novice drivers from experienced drivers^3,11^, they do not provide a theoretical basis to clearly understand driver behavior when faced with hazards. To date, little research has attempted to explain the processes involved in detecting and responding to road hazards using a well-defined theoretical framework^3^. Recently, Barragan and colleagues^12^ have proposed a theoretical framework to provide a better understanding of perceptual attention and behaviors when the driver interacts with hazards. The hazard perception-response framework describes 4 main stages triggered under typical conditions. The first stage, namely *hazard detection*, corresponds to the localization and selective attention to a hazard or a cue (e.g., when a hazard has not yet materialized). In the *hazard awareness* stage, the driver is able to understand the situation and identify whether the situation has a hazardous potential. According to this framework, *hazard perception* corresponds to the process combining the stages of detection and awareness. Then, the *response selection* is the stage during which the driver decides whether an action is necessary and, if so, what type of maneuver to perform to avoid or minimize a potential conflict with the hazard. Finally, the *response* stage refers to the moment where the driver performs an action planned in the previous stage. Within this framework, we define *hazard anticipation* as the process that supports the prediction of future hazards and gives drivers additional time to prepare and execute appropriate driving behavior. Because of conflicting definitions of hazard anticipation^12–14^, we decided in the current study to address hazard anticipation as a process that encompasses the stages of hazard detection, hazard awareness, and response selection.

### Causal relationship between hazard anticipation and stress

We suggested above that hazard anticipation would give drivers more time to prepare an appropriate response. According to the hypothesis of Perkins^15,16^, threat anticipation, through a preparatory response, can reduce or modify the noxiousness of the threat. In this sense, studies conducted in both humans and animals have shown that threat anticipation, induced by a predictable threat (i.e. a threat preceded by a cue), reduces stress^17–22^. Furthermore, research has found that the more urgent the driving situation, the more stressed drivers were^23–25^. Capitalizing on the above information, we hypothesized that hazard anticipation would provide drivers more time to prepare an appropriate response, which would decrease the perceived urgency of the situation and thus induce less stress.

### Hazard anticipation and driving experience

Hazard anticipation and driving experience are interconnected. Numerous research has reported that experienced drivers are better at anticipating hazards than novices^2,26,27^. According to Stahl and colleagues^14^, anticipation skills in more experienced drivers would be superior due to their ability to detect and interpret cues from similar and memorized situations. These better anticipation skills would benefit experienced drivers by allowing them to maximize the time needed to prepare an appropriate response^2^. Based on the assumption that highly experienced drivers have extra time to prepare a response, we hypothesized that they might perceive an actual or potentially hazardous situation as less urgent, and thus experience less stress than less experienced drivers.

### Exploring hazard anticipation and stress in the light of the work of defensive behaviors

Research on defensive behaviors has broadly observed during threat anticipation an archaic defensive behavior: *freezing*^28–30^. Freezing behavior was often seen as a brake on the cardiac and motor systems, thus facilitating threat perception and attention^31–33^. Such a brake in the cardiac system was evidenced by a dominance of parasympathetic over sympathetic activity^34^, and observed through a deceleration of heart rate^28,29,35^. The brake on the motor system was notably deduced by associations, on the one hand, between cardiac deceleration and periaqueductal grey (PAG) responses^34^, well known to be involved in response to threatening situations^36,37^, and on the other hand, between PAG and brain regions involved in motor planning and inhibition. Freezing behavior is also a key stage of threat preparedness, as it corresponds to the moment when the living organism chooses the most appropriate subsequent defensive behavior^38^, such as switching from freezing to flight-or-fight behavior if an escape route is available^30^ or to tonic immobility if the escape route is not available^32^.

The stress response, historically considered as a *flight-or-fight* defensive behavior, was observed by an increase in heart rate indicating a sympathetic discharge and a parasympathetic withdrawal^39–41^. The flight-or-fight behavior corresponds to the stage where the organism moves away from the threat, dodges it laterally, or moves closer to the threat in order to confront it^42^. More recently, a recent study, in which a generalization of this defensive behavior was observed, reported that the increase in heart rate did not reflect only the execution of a motor response (e.g., moving away, moving laterally, or moving closer) but also another component related to stimulus processing^43^.

### The current study

The reported experiment aimed to better understand the processes and driving behaviors involved in hazard anticipation and associated stress, in light of the theory of defensive behaviors. While the theory of defensive behavior has traditionally been used to explain responses and behaviors to threatening stimuli, we argue that this theory could also be used to explain those associated with impending road hazards. In our view, impending road hazards would be similar to threatening stimuli since both are capable of inducing anticipation, stress response and associated motor behaviors (e.g., avoidance behavior).

With this in mind, the current study seeks to determine, first, whether hazard anticipation is triggered in drivers by a cue warning of an upcoming road hazard, second, whether hazard anticipation can mitigate the ensuing stress response, and third, whether the stress response is influenced by driving experience. To answer these questions, three driving scenarios were used such that participants faced a predictable hazard (i.e. a cue followed by a hazard), a false alarm (i.e. a cue only) and an unpredictable hazard (i.e. a hazard only). The cue was provided to trigger hazard anticipation, and a road hazard to induce a stress response. Heart rate and pupil diameter, two physiological measures identified as being sensitive to driver stress^44^, were collected throughout the driving scenarios. In addition to these measures, driving speed as well as subjective levels of stress, arousal and emotions were recorded. The following hypotheses were made:

(i) Hazard anticipation should be triggered by a cue and observed through a freezing behavior—characterized by decrease in heart rate—and an unchanged driving speed. A stress response should be induced by a road hazard (hereafter referred to as the “hazardous event”), and observed by flight behavior—characterized by increase in heart rate—and a reduced driving speed.
(ii) Peak heart rate level as well as levels of reported stress, arousal, and negative emotions should be reduced when the hazardous event is warned, supporting the idea that hazard anticipation—triggered by a cue—plays a beneficial role in mitigating the stress response.
(iii) Peak heart rate level should be reduced in drivers with more driving experience in actual and potentially hazardous situations, suggesting that driving experience influences stress levels due to different hazard anticipation abilities.
(iv) Pupil diameter, revealing mental workload, should be increased as soon as a cue or a hazardous event is detected.

## Methods

### Participants

Thirty-six participants (thirteen women, twenty-three men) aged between 20 and 59 years (M_Age_ = 33.06, SD_Age_ = 11.50) were recruited. They were either students or employees and all were in possession of a driver's license. Driving experience (defined here as the number of years of holding a driver's license) ranged from 1 to 40 years (M_Experience_ = 12.98, SD_Experience_ = 10.10). In terms of driving experience, nineteen were highly experienced drivers (more than 10 years experience, M_Age_ = 40.58, SD_Age_ = 10.94, M_Experience_ = 19.94, SD_Experience_ = 9.04, 42% women) and seventeen were less experienced drivers (less than 10 years experience, M_Age_ = 24.65, SD_Age_ = 3.52, M_Experience_ = 5.20, SD_Experience_ = 3.03, 29% women). The participants had normal or corrected-to-normal vision and declared no cognitive disorders and no heart disease. All participants signed an informed consent form stating that electrocardiogram signals, ocular and driving information were collected throughout the driving simulator experiment. Written informed consent was obtained from all participants. The Ethics Committee of Department of Psychology of Lyon 2 Lumiere approved the study and the methods were carried out in accordance with the relevant guidelines and regulation.

### Experimental design and procedure

**Figure 1:**
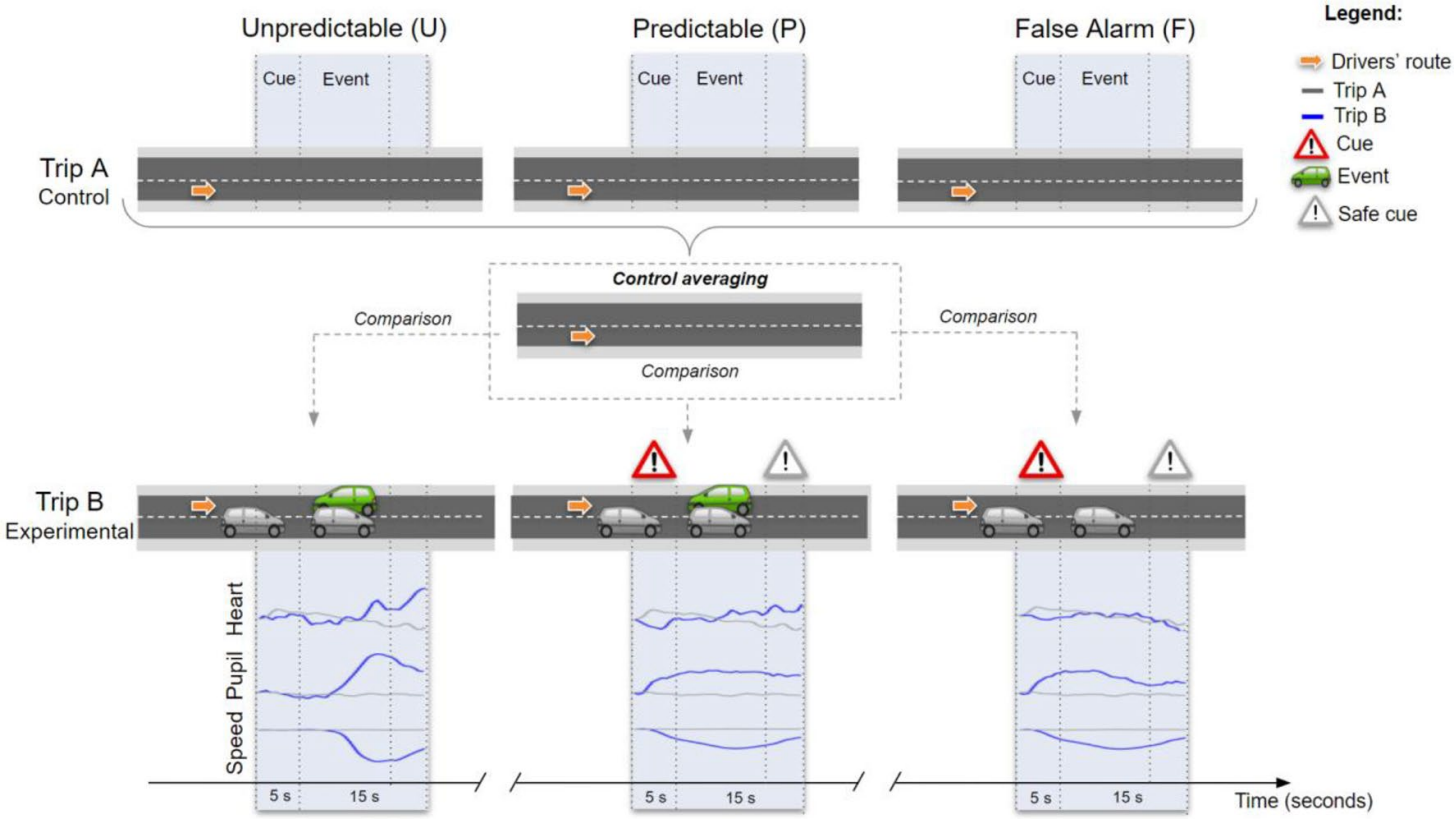
Overview of the drivers’ route in Trip A (control conditions) and Trip B (experimental conditions), as well as the mean changes in heart rate, pupil diameter and driving speed. Experimental conditions were: Unpredictable (U), Predictable (P) and False alarm (F) conditions. Each experimental condition in trip B was compared to the control condition in trip A (i.e., the 3 control conditions averaged), and analyzed over two time windows (post-cue and post-event). The x-axis represents the measurement period for both time windows.

The driving simulator study included three 8-min drives: training, trip A and trip B. For all drives, participants were instructed to comply with traffic laws, follow the GPS directions, and drive and react as they usually would on a real road. No information was given to them about what they will have to deal with while driving. First, participants started with a training drive to discover the driving route and to get familiar with the simulator. Second, they drove on a simulated route without traffic and the trip was recorded to provide control conditions (trip A) (see Fig. 1). Third, they drove on the same simulated route as trip A but this time with traffic and under experimental conditions (trip B). All participants faced three experimental conditions: a safe condition (False alarm; F) involving a hazard cue with no hazardous event, and two hazardous conditions—one in which the hazardous event occurs unpredictably (Unpredictable; U), and the other in which the hazardous event is signaled by a hazard cue (Predictable; P). It should be noted that the choice of cue and event was inspired by a recent study^45^ in which hazard anticipation and stress were successfully manipulated using this same type of cue and event:

- *Hazard cue* The cue was depicted by an alert message that popped up on a simulated phone located on the windshield. For the P and F conditions, the cue was visible to drivers (i.e., Cue ON) until the hazard appeared, in order to generate hazard anticipation. In contrast, no cue was delivered to drivers in the condition U, so that the hazardous event was unexpected.
- *Hazardous event* The hazardous event was represented by an oncoming car approaching on the wrong side of the road (i.e., same driving lane as the participants’ vehicle). For the P and U conditions, the event was visible (i.e., Event ON). However, in the condition F, the event did not manifest so as to study only the effect of the hazard cue. Once the event was passed by the driver, a safe cue popped up on the simulated phone as for the hazard cue. Safe cue was provided in the conditions P and U, but also in the condition F so that drivers could stop waiting for a hazard that never came.

As the cues and hazardous events in the experimental conditions were the same, the order of experimental conditions was counterbalanced within trip B. Thus, drivers were equally distributed within each combination group: UPF, UFP, PUF, PFU, FPU, FUP. Within these combination groups, the order of trip A and trip B was also counterbalanced to mitigate learning and acclimatization effects related to the driving simulator^46^.

During experimental and control conditions, data from heart rate, pupil diameter and driving speed were gathered. The collected data were used to explore hazard anticipation in the post-cue time window, and the stress response in the post-event time window, by comparing the three experimental conditions (U, P and F) to the control conditions (same road locations in trip A). As the control situations were the same (i.e., without cue, event and traffic), the three control conditions were pooled into a single baseline before being compared to each experimental condition. At the end of the experiment, drivers were asked to report levels of stress, arousal and valence for the conditions U, P and F. Reported stress was assessed using a 5-point Likert stress scale, ranging from 1 "not stressful at all" to 5 "extremely stressful". Reported arousal and valence were rated using a Self-Assessment Manikin (SAM) questionnaire^47^ including scales from − 2 to 2 (e.g., 2 for valence dimension indicates positive emotions, and 2 for arousal dimension reports highest arousal).

### Apparatus

**Figure 2:**
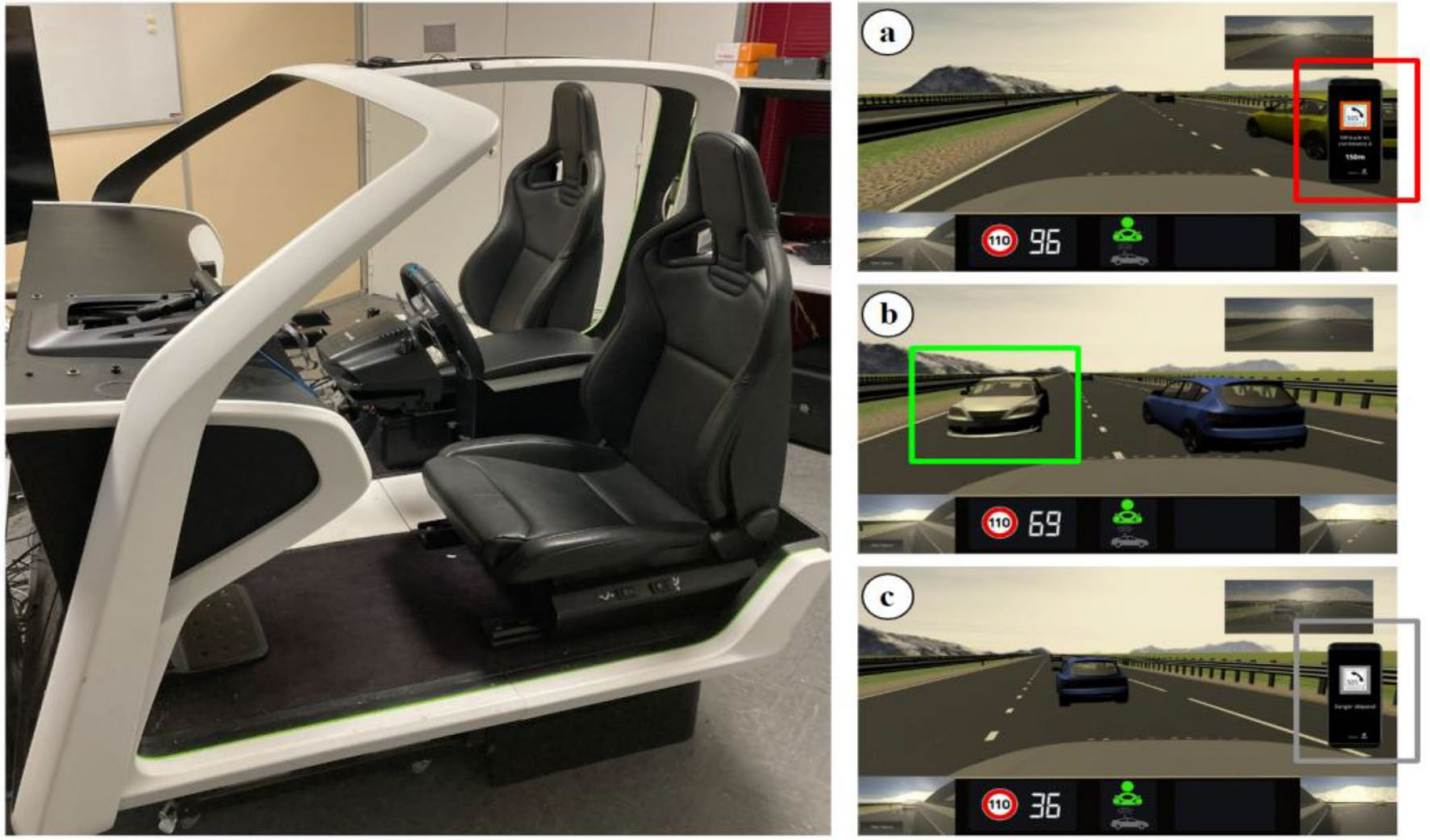
Overview of the homemade driving simulator and driving scenarios designed on Unity 3D software: (**a**) a hazard cue, displayed on the phone, is provided to the driver, (**b**) a hazardous event on the wrong side of the road approaches the driver, (**c**) a safe cue, displayed on the phone, is provided to the driver after the event has passed.

The experiment was performed in a homemade, fixed-base driving simulator (see Fig. 2) with a fully equipped interior: automatic gearbox, steering wheel and pedals (Logitech G29). Simulation environment, rear-view mirror and side mirrors were displayed on a screen (16:9) in front of the simulator. Unity 3D software was used to design the simulated driving environment. A physiological data acquisition system (BIOPAC MP160) was set up to collect drivers’ cardiac responses with a sampling rate of 500 Hz. Drivers’ ocular responses were collected via an eye tracker (FOVIO) with a sampling rate of 60 Hz. A calibration was performed for each driver prior to the training drive. Driving speed and simulation-related data (e.g., time markers for cues and hazardous events) were collected at a sampling rate of 10 Hz. Finally, Rtmaps software was used to time-stamp, record, and synchronize the data from the different sensors.

### Measurement and data processing

#### Heart rate

Cardiac data were pre-processed as follows: (1) a band-pass filter between 2 and 40 Hz was applied on the electrocardiogram signals to remove signal drifts, (2) R–R peaks were detected using an automatic detection procedure (AcqKnowledge 5.0 software) then extracted after a visual check of the ECG signals for artifacts and correction for misplaced or omitted R waves, (3) the R–R intervals were converted into heart rate (beats per minute), (4) the heart rate data was sampled every 0.5 s for a period of − 0.5 up to 4.5 s after cue onset (i.e. 10 time points), and − 0.5 up to 14.5 s after event onset (i.e. 30 time points). The heart rate value in the last half-second prior to cue onset and event onset was used as a baseline for the post-cue and post-event heart rate values, respectively. Data sampling was performed using a cubic spline interpolation. (5) Finally, heart rate change was calculated by subtracting the baseline from each post-baseline heart rate value.

#### Pupil diameter

Pupil data pre-processing consisted of: (1) averaging the data from both eyes to obtain one value for each participant at any moment, (2) sampling pupil data every 0.5 s over two time windows (post-cue and post-event) in the same way as for heart rate, (3) normalization of the pupil data for each driver, according to her/his average pupil diameter over the three drives, (4) subtracting the baseline value (i.e., at − 0.5 s after cue or event onset) from each post-baseline pupil data value.

#### Driving speed

Speed data were converted to speed change using the same methodology as for heart rate and pupil diameter, i.e., sampling data every 0.5 s over two time windows and subtracting the baseline from each post-baseline speed value.

#### Self-assessment

Reported stress, arousal and valence scores were normalized for each driver by computing the z-score as follows: z = (x − $\mu$)/$\sigma$ where:z = the standard score for a random driver,x = the reported score by the driver,$\mu$ = the mean of all reported scores by the driver (at rest, in trip A, in trip B), and $\sigma$ = the standard deviation of all reported scores by the driver.

### Statistical analysis

The analyses of patterns reflecting hazard anticipation and stress were carried out separately. To explore hazard anticipation first, cardiac, pupil and driving speed responses in the post-cue time window were analyzed using two-way repeated measures ANOVAs. The analyses included as within-subject factors the condition (U vs. control, P vs. control, F vs. control) and time point (10 levels). Second, to investigate stress, cardiac, pupil and driving speed responses were examined in the post-event time window using two-way repeated measures ANOVAs. These analyses included as within-subject factors the condition (U vs. control, P vs. control, F vs. control) and time point (30 levels). For all analyses, degrees of freedom were adjusted according to the Greenhouse–Geisser method to compensate for violations of the sphericity assumption. ANOVAs indicating a significant condition × time interaction led to planned comparisons for each time point between the control condition and the condition U, P or F. When ANOVAs showed no interaction but a significant main effect of condition, planned comparisons were carried out between the control condition and the condition U, P or F, all averaged over the time window.

In order to study whether hazard anticipation has a beneficial effect for the subsequent stress response, we calculated a Linear Mixed Model (LMM) on the peak heart rate levels, in conditions U, P and F. Similar to a previous research^43^, we first retained, for each participant, the maximum value of heart rate, specifically in the post-event time window. We then averaged the maximum values of heart rate for each condition. The LMM analysis was also used to determine whether the stress response was influenced by driving experience. Accordingly, level of driving experience (i.e. higher experience and lower experience) and condition type (i.e. U, P and F) were both included in fixed effects. In order to account for a general variability of cardiac response across drivers, participant ID was included in random effects. The model was fitted using the Restricted Maximum Likelihood technique. *P*-values were derived based on Satterthwaite’s approximation of degrees of freedom. The LMM analysis, performed above with heart rate, was then repeated on the reported stress, valence and arousal scores. All analyses in this study were carried out using JASP (Version 0.16.3) and the significance level α was set at *p* < 0.05.

## Results

### Exploring patterns of hazard anticipation and stress

**Figure 3:**
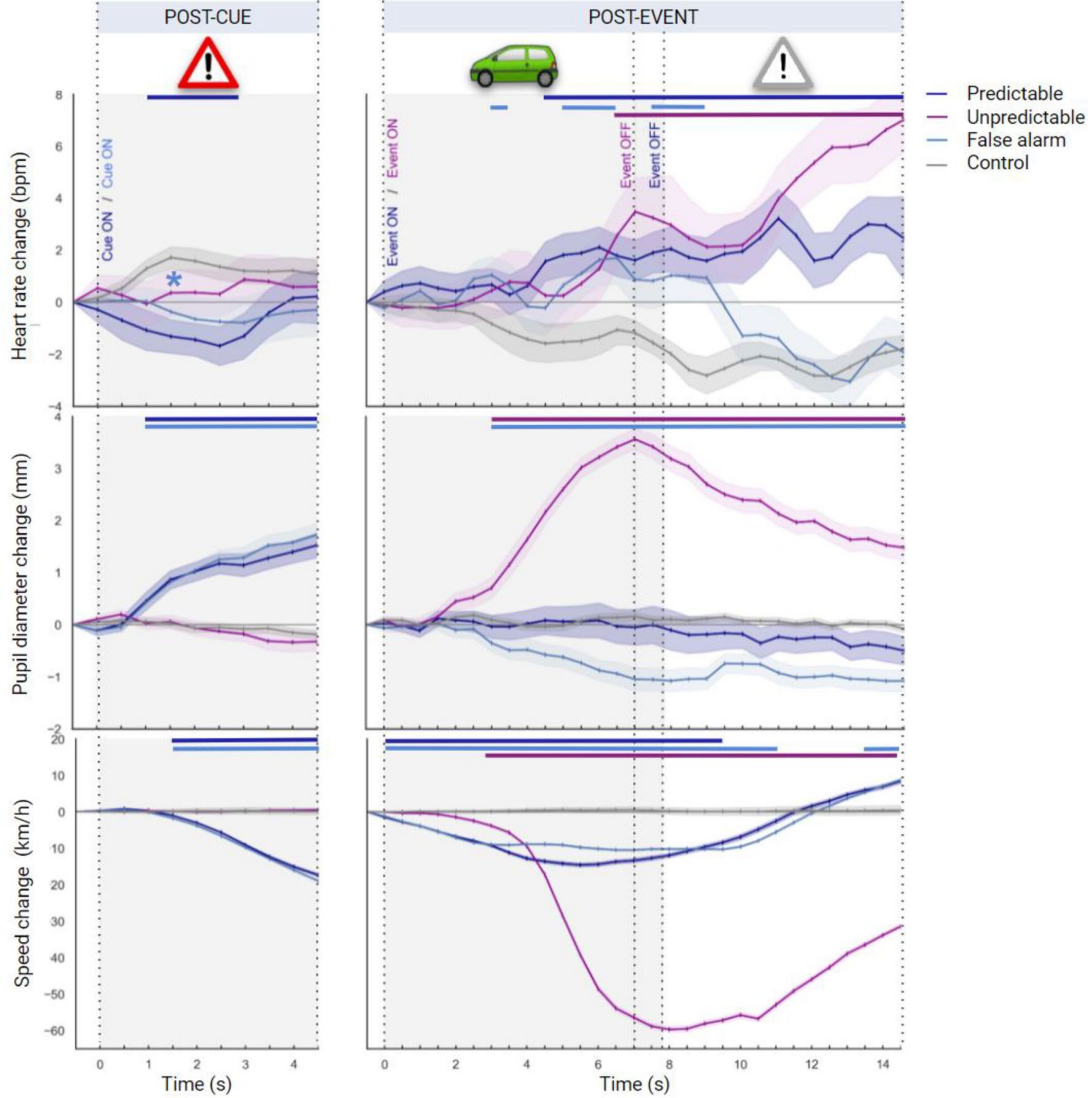
Changes in heart rate, pupil diameter and driving speed relative to the last half-second prior to cue-onset and event-onset when drivers experienced the control (gray), the false alarm (light blue), the predictable (navy blue) and the unpredictable (purple) conditions. Cues and events were either ON (visible) or OFF (invisible). Shaded areas denote standard errors of the mean. Time points with significant differences between the control and experimental conditions are displayed at the top of each figure as a colored horizontal line. When interaction was not significant, only the significant main effect of condition was displayed and represented by an asterisk: * (i.e. *p* < .05). The pictograms at the top of the figures indicate cue ON, event ON and cue OFF respectively.

In order to investigate hazard anticipation, two-way repeated-measures ANOVAs were run, for each measure, in the post-cue time window. Conditions (U vs. control, P vs. control, F vs. control) and time point (10 levels) were included in the ANOVAs as within-subject factors (see Fig. 3, and Table 1 for complete ANOVA results).

**Table 1:** ANOVA outcomes for physiological and driving speed responses in the post-cue time window.

Response	Control vs	Condition			Time window			Condition × Time window		
		*F*	(df)	*n*^*2*^p	*F*	(df)	*n*^*2*^p	*F*	(df)	*n*^*2*^p
Heart rate	False alarm	4.0*	(1, 35)	0.105	0.43	(2.58, 90.34)	0.012	1.2	(2.24, 32.82)	0.035
	Predictable	6.5*	(1, 35)	0.157	0.90	(3.28, 114.8)	0.025	2.7*	(3.01, 105.6)	0.074
	Unpredictable	0.45	(1, 35)	0.013	0.87	(2.85, 100.0)	0.024	0.66	(3.07, 107.5)	0.019
Pupil diameter	False alarm	32.2***	(1, 35)	0.480	33.3***	(2.93, 102.7)	0.488	41.3***	(2.62, 91.9)	0.541
	Predictable	26.2***	(1, 35)	0.428	**20.6**	(3.57, 125.1)	0.371	28.9***	(2.66, 93.3)	0.453
	Unpredictable	0.17	(1, 35)	0.005	6.2***	(3.57, 125.1)	0.152	1.3	(3.83, 134.1)	0.036
Driving speed	False alarm	45.6***	(1, 35)	0.566	54.4***	(1.43, 50.2)	0.609	61.7***	(1.51, 52.9)	0.638
	Predictable	29.6***	(1, 35)	0.459	38.8***	(1.40, 49.2)	0.526	32.6***	(1.32, 46.2)	0.482
	Unpredictable	0.025	(1, 35)	0.000	0.14	(1.28, 44.8)	0.004	0.31	(1.48, 51.9)	0.009

*F* (df) = statistics of ANOVA and degrees of freedom (in parentheses). *p < .05. **p < .01. ***p < .001.

Significant values are in bold.

#### Heart rate

Planned comparisons indicated that heart rate was significantly lower in the condition P compared to the control condition, from 1 to 3 s in the post-cue window, and significantly lower in the condition F compared to the control condition overall in the post-cue window [*t* = − 2.021, *p* < 0.05*] (see colored lines above the graph in Fig. 3, and "Supplementary Material" for statistics of planned comparisons).

#### Pupil diameter

Pupil diameter was significantly larger in the condition P compared to the control condition, and in the condition F compared to the control condition, both from 1 s to 4.5 s in the post-cue window.

#### Driving speed

Driving speed was significantly lower in the condition P compared to the control condition, and in the condition F compared to the control condition, both between 1.5 s and 4.5 s in the post-cue window.

Then, to explore stress, two-way repeated-measures ANOVAs were run for each measure in the post-event time window. Conditions (U vs. control, P vs. control, F vs. control) and time point (30 levels) were included in the ANOVAs as within-subject factors (see Fig. 3, and Table 2 for full ANOVA results).

**Table 2:** ANOVA outcomes for physiological and driving speed responses in the post-event time window.

Response	Control vs	Condition			Time window			Condition × Time window		
		*F*	(df)	*n*^*2*^p	*F*	(df)	*n*^*2*^p	*F*	(df)	*n*^*2*^p
Heart rate	False alarm	3.4	(1, 35)	0.090	6.0***	(6.30, 220.7)	0.148	2.4*	(6.41, 224.6)	0.067
	Predictable	12.8***	(1, 35)	0.268	0.78	(7.78, 272.3)	0.022	4.2***	(6.75, 236.2)	0.109
	Unpredictable	14.8***	(1, 35)	0.298	3.6**	(5.36, 187.7)	0.093	9.3***	(5.52, 193.3)	0.212
Pupil diameter	False alarm	22.0***	(1, 35)	0.387	11.0***	(5.85, 204.7)	0.240	9.9***	(5.76, 201.8)	0.221
	Predictable	0.6	(1, 35)	0.018	1.5	(3.97, 139.0)	0.042	0.99	(3.69, 129.3)	0.028
	Unpredictable	116.6***	(1, 35)	0.769	54.9***	(4.70, 164.7)	0.611	50.2***	(4.35, 152.5)	0.589
Driving speed	False alarm	20.8***	(1, 35)	0.373	29.2***	(3.27, 114.7)	0.455	29.0***	(2.83, 99.3)	0.453
	Predictable	6.2*	(1, 35)	0.151	10.3***	(2.19, 76.7)	0.228	10.2***	(1.99, 69.8)	0.226
	Unpredictable	162.7***	(1, 35)	0.823	55.5***	(2.42, 84.7)	0.614	54.5***	(2.39, 83.6)	0.609

*F* (df) = statistics of ANOVA and degrees of freedom (in parentheses). *p < .05. **p < .01. ***p < .001.

Significant values are in bold.

#### Heart rate

Planned comparisons indicated that heart rate was significantly greater in the condition U compared to the control condition from 6.5 s in the post-event window, in the condition P compared to the control condition from 4.5 s, and in the condition F compared to the control condition from 3 s to 3.5 s, then from 5 s to 6.5 s, and from 7.5 s to 9.5 s (see colored lines above the graph in Fig. 3, and "Supplementary Material" for statistics of planned comparisons).

#### Pupil diameter

Pupil diameter was significantly larger in the condition U versus the control condition from 3 s in the post-event window. In contrast, the pupil diameter significantly reduced in the condition F *versus* the control condition from 3 s. Interestingly, no change in pupil diameter was observed between the condition P and the control condition.

#### Driving speed

Driving speed was significantly lower in the condition U compared to the control condition from 3 s in the post-event window, in the condition P compared to the control condition from 0 s to 9.5 s, and in the condition F compared to the control condition from 0 s to 11.5 s and from 13.5 s to 14.5 s.

### Effect of hazard anticipation and driving experience on stress

**Figure 4:**
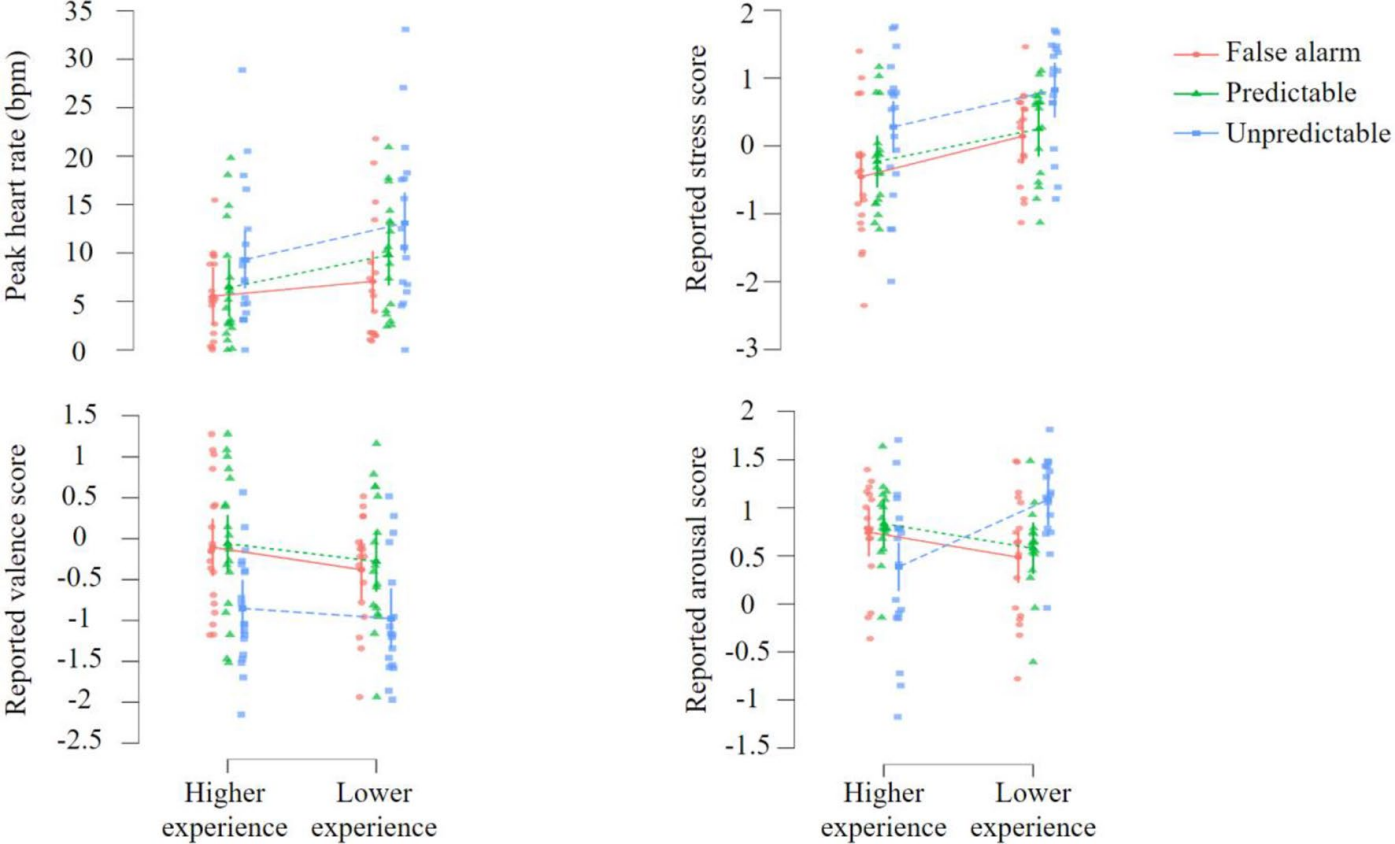
Scatter plots depicting means of peak heart rate, reported stress, reported valence and reported arousal, as a function of condition type (False alarm, Predictable and Unpredictable) and level of driving experience (higher and lower). Each point represents the value of one participant.

The effects of hazard anticipation and driving experience on stress were explored using LMM analyses (see Fig. 4, Table 3 for results of LMM, and "Supplementary Material" for the raw descriptive statistics of dependent variables).

**Table 3:** Results of Linear Mixed Model analyses for the dependent variables: main effects of condition, driving experience and interaction effect of condition and driving experience.

	Condition	Driving experience	Condition × Driving experience
	*F (df)*	*F (df)*	*F (df)*
Peak heart rate	6.177 (2, 68)*	3.888 (1, 34)	0.363 (2, 68)
Reported stress	11.133 (2, 68)***	6.452 (1, 34)*	0.069 (2, 68)
Reported valence	21.607 (2, 68)***	0.947 (1, 34)	0.184 (2, 68)
Reported arousal	0.480 (2, 68)	0.280 (1, 34)	9.436 (2, 68)***

*p < .05; **p < .01; ***p < .001.

#### Peak heart rate

Results of the LMM analysis revealed a significant main effect of condition type, but no main effect of driving experience and interaction between condition type and driving experience (Table 3). Pairwise comparisons then indicated that the level of peak heart rate was higher in condition U than in condition F (*t*(68) = 3.475, *p* < 0.001, 95% CI [2.084, 7.704]) and condition P (*t*(68) = 2.193, *p* < 0.05, 95% CI [0.278, 5.898]) (Fig. 4). However, the peak heart rate level was similar between conditions F and P (*t*(68) = 1.282, *p* > 0.05, 95% CI [− 1.004, 4.616]).

#### Subjective stress

The LMM analysis reported significant main effects of condition type and driving experience, but no interaction between condition type and driving experience (Table 3). Indeed, the level of reported stress was higher in the condition U compared to condition F (*t*(68) = 4.509, *p* < 0.001, 95% CI [0.395, 1.023]) and condition P (*t*(68) = 3.459, *p* < 0.001, 95% CI [0.230, 0.858]) (Fig. 4). In contrast, the reported stress level was unchanged between conditions F and P (*t*(68) = 1.050, *p* > 0.05, 95% CI [− 0.149, 0.479]). Moreover, drivers with higher experience reported less stress than those with lower experience (*t*(34) = − 2.450, *p* < 0.05, 95% CI [− 0.974, − 0.108]).

#### Subjective valence

The results obtained from the LMM analysis indicated a significant main effect of condition type, but no main effect of driving experience and interaction between condition type and driving experience (Table 3). Then, pairwise comparisons revealed that the level of reported valence was significantly lower for condition U compared to condition F (*t*(68) = − 5.381, *p* < 0.001, 95% CI [− 0.922, − 0.423]) and condition P (*t*(68) =  − 5.961, *p* < 0.001, 95% CI [− 0.995, − 0.496]) (Fig. 4). In addition, no change in the reported valence level was observed between conditions F and P (*t*(68) = 0.580, *p* > 0.05, 95% CI [− 0.177, 0.322]).

#### Subjective arousal

The LMM analysis reported a significant interaction between condition type and driving experience (Table 3). In contrast, no main effects were found for condition type and driving experience. Pairwise comparisons then showed that drivers with higher experience reported similar levels of arousal than drivers with lower experience in both conditions F (*t*(101.231) = 1.408, *p* > 0.05, 95% CI [− 0.107, 0.631]) and P (*t*(101.231) = 1.381, *p* > 0.05, 95% CI [− 0.112, 0.626]) (Fig. 4). However, while more experienced drivers reported being less aroused in condition U than in condition F (*t*(68) = − 2.060, *p* < 0.05, 95% CI [− 0.711, − 0.011]) and condition P (*t*(68) = -2.562, *p* < 0.05, 95% CI [− 0.799, − 0.099]), less experienced drivers reported to being more aroused in the condition U than in condition F (*t*(68) = 3.243, *p* < 0.01, 95% CI [0.231, 0.970]) and condition P (*t*(68) = 2.742, *p* < 0.01, 95% CI [0.138, 0.877]).

## Discussion

Our results showed that drivers only exhibited a clear cardiac deceleration when a hazard cue was provided. This observation is in line with previous studies reporting *fear bradycardia*^48,49^ during threat anticipation and interpreted as a parasympathetic physiological indicator of freezing behavior^38,50^. Based on previous work exploring freezing with active coping opportunities in driving^45^ and non-driving contexts^28,30^, the freezing behavior expressed by the drivers in this study could be considered as a state of “active preparation” for subsequent flight behavior. In the same way that freezing should not be confined to a purely passive state, a recent research reported that a state of preparedness, similar to freezing, facilitated perceptual decision^43^. Indeed, the authors found greater anticipatory cardiac deceleration during a more complex decision task. Applying this finding to our work, this would mean that hazard anticipation induced by hazard predictability, would support drivers in decision-making processes. Freezing behavior was then followed by flight behavior in the false alarm and predictable conditions, identified by a gradual increase in heart rate in the post-event time window. These flight behaviors can legitimately be qualified as "anticipatory" stress responses since they occur a few seconds earlier as compared to those recorded in the unpredictable condition. In addition, from a behavioral perspective, the anticipatory stress responses could also be evidenced by early and progressive decelerations of speed, reflecting avoidance behavior. By contrast, a speed deceleration in the unpredictable condition appeared later and abruptly. Collectively, these findings support that hazard anticipation and anticipatory stress response can be successfully detected through temporally and spatially distinct cardiac and behavioral patterns.

Our hypothesis, that hazard anticipation would reduce driver stress, was validated by the results of comparing the false alarm and predictable conditions—where freezing was evidenced—to the unpredictable condition—where no freezing was diagnosed. Indeed, drivers expressed reduced flight behavior (i.e., positive heart rate peak was lower), and reported less stress and negative emotions, in the false alarm and predictable conditions than in the unpredictable condition. This finding converges with previous automotive research that extensively reported distress while driving^51–57^, i.e. the variant of stress highly related with negative emotions. Furthermore, previous research argued that stress, arousal and performance were related and that this relationship could be described by the so-called inverted-U shaped curve established by Yerkes and Dodson^58^. Moreover, Cohen’s synthesis states that the most popular arousal theory suggests that individuals confronted with unpredictable stressors show higher levels of arousal^18^. In contrast, in the current study, drivers reported more stress in the unpredictable condition but not more arousal, which is somewhat puzzling. However, by dividing the sample of drivers by level of driving experience into two groups, less experienced drivers reported higher levels of arousal in the unpredictable condition, whereas more experienced drivers reported lower levels of arousal in the same condition. While the stress and arousal scores reported by less experienced drivers follow the logic that the more stressed drivers are, the more aroused they are, this is not the case for scores reported by more experienced drivers. Further investigations would be needed to better understand why more experienced drivers reported lower levels of arousal for the condition they rated as the most stressful, i.e. the unpredictable condition.

Based on the assumption that more experienced drivers have additional time to prepare a response, we hypothesized that they might perceive an actual or potentially hazardous situation as less urgent, and thus experience less stress than less experienced drivers. The results partially support this theory, as drivers with higher experience reported lower levels of stress than drivers with lower experience. However, peak heart rate levels did not reach statistical significance, so this hypothesis could not be validated on a physiological level based on the reported findings.

In the current study, pupil dilation was observed after each onset of a hazard cue or a hazardous event. The fact that a gradual decrease in pupil diameter was found for the false alarm condition (including only a hazard cue) after a few seconds in the post-event time window suggests that once drivers were no longer stimulated by a new stimulus from the environment, pupil diameter inevitably decreased. More interesting, the safe cue—indicating that hazard was exceeded—did not lead to a re-increase in pupil diameter in the false alarm condition, while it was also a new stimulus providing information. It can be hypothesized that in a threatening driving context, only a sufficiently relevant stimulus is able to keep the pupil dilated. The fact that the drivers' pupils kept dilated after the occurrence of the predictable hazardous event supported this idea, as it meant that the event was sufficiently relevant to warrant sustained alertness up to the safe cue onset. In addition, the fact that drivers' pupils gradually decreased when the hazardous event was exceeded confirmed the relationship previously reported in a driving simulator study between pupil dilation and conscious detection of road hazard^59^. However, conversely to this study who showed pupil dilation when hazardous road events occurred, and as mentioned above, we also observed pupil dilation after a hazard cue, i.e. when a hazardous event was expected. Again, our result is in line with previous non-automotive research in which pupil dilation was accompanied by bradycardia during anticipation of threatening^34,60^ and non threatening stimuli^61^. Finally, the fact that pupil dilation and cardiac deceleration were found during hazard anticipation is consistent with a previous claim that there is an increased need to take in visual information in potentially threatening situations^62^.

In hindsight, the present work might provide an effective contribution to a broader literature related to orienting attention. Indeed, orienting attention is typically explored by the “Spatial Orienting Paradigm”^63,64^ involving a valid cue and an invalid cue, each presented before an impending stimulus. While the valid cue directs attention to a location where something relevant will appear, the invalid cue directs attention to a location where nothing relevant will appear. On this basis, a parallel can be drawn between the work conducted on orienting attention—using the Spatial Orienting Paradigm—and the work of the present study—using a revised paradigm adapted to a driving context. Indeed, the cue presented in the Predictable condition can be considered a valid cue while the cue presented in the False Alarm condition can be regarded as an invalid cue. As mentioned above, both cues in the Predictable and False Alarm conditions induced freezing behavior during hazard anticipation. Based on the work on defensive behaviors, exploring freezing behavior would be a privileged window for studying driver attention, as this behavior, in particular, would facilitate orienting selective attention to the threat or expected threat^31–33^. Furthermore, a recent study, in which the Spatial Orienting Paradigm was also adapted to the driving context, replicated the effects of orienting attention—repeatedly observed in the laboratory—on hazard prediction performance^65^. Indeed, Muela and colleagues found that the invalid cues resulted in poorer scores of hazard prediction than the valid cues^65^. Based on our results and the literature discussed, it appears clear that the use of a Spatial Orienting Paradigm adapted to the driving context, combined with the use of different measures (e.g., performance, physiological, behavioral), constitutes a relevant methodology to better understand the implications and manifestations of driver attention during hazardous situations.

Previous research has reported faster responses for abrupt-onset hazards (1.79 s), i.e., hazards that capture attention through their salience in the road environment and the risk of imminent collision, than for gradual-onset hazards (3.87 s), i.e., hazards that capture attention through the use of environmental cues^66^. After observing similar results, another research concluded that abrupt-onset hazards attract attention faster than gradual-onset hazards. The authors then suggested that for abrupt-onset hazards, there would not be enough time to take advantage of environmental cues^67^. To improve hazard detection performance, particularly for abrupt-onset hazards, the authors investigated the effects of driving experience and proactive commentary training (in which verbal information was provided to drivers to allocate attention directly to the most appropriate location in the driving scene, thereby saving time to better take advantage of environmental cues). Their results indicate that driving experience would have only a limited effect on improving performance in detecting abrupt-onset hazards. However, proactive commentary training would have a major and positive effect on performance by allowing drivers to anticipate the hazard. Furthermore, in the current study, the findings revealed that the anticipation of abrupt-onset hazards could also be triggered by a cue warning. Consequently, our results collected, along with those of Castro and colleagues^67^, demonstrate the value of using both cue warning and proactive commentary training to facilitate the anticipation of abrupt-onset hazards while driving.

## Limitations

In the current study, the control condition showed highly fluctuating cardiac changes over time. Unlike the majority of studies that examined defensive behaviors, this study explored these behaviors in a simulated driving environment in which drivers were virtually moving. As a result, the driving environment (e.g., road architecture, surrounding cars) could substantially affect the cardiac response. We believe that it was precisely the road architecture that influenced the drivers' control condition. Indeed, all cues were delivered a few seconds after exiting a turn. This hypothesis is further reinforced by the fact that the study that inspired our research^45^ did not show such cardiac fluctuations in the control condition, probably because the exploration of the cardiac response was performed in a straight line, away from any turns. In addition, because simulator driving was a relatively new activity, cognitive and motor efforts might have been magnified due to poor vehicle control in the simulated environment. Although the novelty effect related to the simulator was attempted to be reduced by the training phase, a novelty effect probably persisted. Given the cardiac fluctuation within the control condition, we chose to compare each experimental condition with the control condition in order to remove the environment-related effects. Therefore, our results remain unchanged and cannot be affected by the changing environment.

Furthermore, this study drew conclusions on the basis of driver responses collected in a driving simulator. Assuming that setting conditions, real or simulated driving, affect physiological measures, it would be worthwhile to also examine drivers' defensive behaviors on real roads so as to gauge any potential differences.

To broaden the understanding of processes and behaviors in hazard anticipation and stress, we encourage future studies to use a similar methodology to explore other cues and road hazards. Previous studies have examined different hazardous situations using a similar methodology; however, they are still few in number^45,68^. We also encourage the exploration of cues, for example, by varying the duration of their presentation to drivers. Indeed, previous research has shown that the duration of cue presentation on the screen influences drivers' anticipation abilities^2^.

Regarding the effect of driving experience, it cannot be excluded that the relatively small sample sizes in this study (19 highly experienced drivers versus 17 less experienced drivers) could have failed to reveal an effect of driving experience on the physiological responses due to high inter-individual variability. In addition, it should be noted that the sample in this study was composed mostly of experienced (M_Experience_ = 5.20 years) and highly experienced drivers (M_Experience_ = 19.94 years). It would therefore be interesting in future studies to compare experienced and highly experienced drivers to novice drivers. Since many studies have reported that novice drivers are poorer at anticipating hazards^2,26,27^ and overrepresented in crashes, especially in the first twelve month after licensure in comparison to experienced drivers^69–73^, we assume that clearer differences in responses and behaviors might be identified between experienced or highly experienced drivers and novice drivers. Furthermore, while the number of years licensed was used as the sole criterion in this study to reflect the level of driving experience, future studies should supplement this criterion with the number of kilometers or miles driven, so that the effect of driving experience can be investigated more accurately.

Despite these limitations, this study shows how previous work on defensive behaviors can be used to gain insight into the processes and driving behaviors involved in hazard anticipation and stress. On a practical level, this new insight could be useful for studying the effects of warnings delivered while driving. This in-depth exploration would then make it possible to adapt warnings, for example, according to the level of driving experience, so that drivers would be better at anticipating hazards and would experience limited stress.

## Supplementary Information

Supplementary Information.

## Data Availability

The datasets analyzed during the current study are available from the corresponding author upon reasonable request.
