# Supplementary material for: Exploring hazard anticipation and stress while driving in light of defensive behavior theory
Source: Sci Rep. 2023 May 15;13:7883. doi: 10.1038/s41598-023-34714-7 (PMC10185512; doi:10.1038/s41598-023-34714-7)
Supplement: Supplementary file 1 — Supplementary Information. [file 41598_2023_34714_MOESM1_ESM.docx]

**Exploring hazard anticipation and stress while driving in light of defensive behavior theory**

Laora Kerautret^a,b*^, Stephanie Dabic^a^, Jordan Navarro^b,c^

^a^ Valeo Interior Controls, Rue Jules Verne, 74100, Annemasse, France

^b^ Laboratoire d’Etude des Mecanismes Cognitifs (EA 3082), University Lyon 2, 5 avenue Pierre Mendès, Bron, 69676, France

^c^ Institut Universitaire de France, Paris, France

E-mail addresses: [laora.kerautret@univ-lyon2.fr](mailto:laora.kerautret@univ-lyon2.fr) (L. Kerautret), [stephanie.dabic@valeo.com](mailto:stephanie.dabic@valeo.com) (S. Dabic), [jordan.navarro@univ-lyon2.fr](mailto:jordan.navarro@univ-lyon2.fr) (J. Navarro)

*Corresponding author: Laora Kerautret will handle correspondence at all stages of refereeing and publication.

#### Supplementary Material

##### Table S1. Heart rate

|  |  | Post-cue window | | |  | Post-event window | | |
| --- | --- | --- | --- | --- | --- | --- | --- | --- |
| Time (s) | Statistics | False alarm  vs. Control | Predictable  vs. Control | Unpredictable  vs. Control |  | False alarm  vs. Control | Predictable  vs. Control | Unpredictable  vs. Control |
| 0 | t | - | -0.883 | - |  | 0.262 | -0.807 | 0.041 |
|  | p | - | 0.383 | - |  | 0.795 | 0.425 | 0.967 |
| 0.5 | t | - | -1.496 | - |  | -0.296 | -0.939 | 0.098 |
|  | p | - | 0.144 | - |  | 0.769 | 0.354 | 0.922 |
| 1 | t | - | -2.691 | - |  | -0.777 | -1.144 | -0.006 |
|  | p | **-** | **< .05*** | - |  | 0.442 | 0.260 | 0.995 |
| 1.5 | t | - | -3.729 | - |  | -0.284 | -1.113 | -0.095 |
|  | p | - | **< .001***** | - |  | 0.778 | 0.273 | 0.925 |
| 2 | t | - | -3.672 | - |  | -0.518 | -0.987 | -0.221 |
|  | p | - | **< .001***** | - |  | 0.607 | 0.330 | 0.826 |
| 2.5 | t | - | -3.331 | - |  | -1.941 | -1.281 | -0.593 |
|  | p | **-** | **< .01**** | - |  | 0.060 | 0.209 | 0.557 |
| 3 | t | - | -2.451 | - |  | -2.645 | -1.622 | -1.004 |
|  | p | - | **< .05*** | - |  | **< .05*** | 0.114 | 0.322 |
| 3.5 | t | - | -1.389 | - |  | -2.357 | -1.279 | -1.215 |
|  | p | - | 0.174 | - |  | **< .05*** | 0.209 | 0.233 |
| 4 | t | - | -0.810 | - |  | -1.519 | -1.626 | -1.314 |
|  | p | - | 0.423 | - |  | 0.138 | 0.113 | 0.197 |
| 4.5 | t | - | -0.516 | - |  | -1.409 | -2.543 | -1.190 |
|  | p | - | 0.609 | - |  | 0.168 | **< .05*** | 0.242 |
| 5 | t | - | - | - |  | -2.080 | -2.608 | -1.190 |
|  | p | - | - | - |  | **< .05*** | **< .05*** | 0.242 |
| 5.5 | t | - | - | - |  | -2.463 | -2.745 | -1.503 |
|  | p | - | - | - |  | **< .05*** | **< .01**** | 0.142 |
| 6 | t | - | - | - |  | -2.792 | -3.277 | -1.791 |
|  | p | - | - | - |  | **< .01**** | **< .01**** | 0.082 |
| 6.5 | t | - | - | - |  | -2.655 | -2.960 | -2.429 |
|  | p | - | - | - |  | **< .05*** | **< .01**** | **< .05*** |
| 7 | *t* | - | - | - |  | -1.899 | -2.947 | -3.087 |
|  | *p* | - | - | - |  | 0.066 | **< .01**** | **< .01**** |
| 7.5 | *t* | - | - | - |  | -1.993 | -3.646 | -2.898 |
|  | *p* | - | - | - |  | **< .05*** | **< .001***** | **< .01**** |
| 8 | *t* | - | - | - |  | -2.582 | -3.615 | -2.665 |
|  | *p* | - | - | - |  | **< .05*** | **< .001***** | **< .05*** |
| 8.5 | *t* | - | - | - |  | -3.302 | -3.762 | -3.469 |
|  | *p* | - | - | - |  | **< .01**** | **< .001***** | **< .001***** |
| 9 | *t* | - | - | - |  | -3.108 | -3.597 | -3.381 |
|  | *p* | - | - | - |  | **< .01**** | **< .001***** | **< .01**** |
| 9.5 | *t* | - | - | - |  | -1.919 | -3.234 | -3.312 |
|  | *p* | - | - | - |  | 0.063 | **< .01**** | **< .01**** |
| 10 | *t* | - | - | - |  | -0.749 | -2.862 | -3.117 |
|  | *p* | - | - | - |  | 0.459 | **< .01**** | **< .01**** |
| 10.5 | *t* | - | - | - |  | -0.628 | -3.261 | -3.320 |
|  | *p* | - | - | - |  | 0.534 | **< .01**** | **< .01**** |
| 11 | *t* | - | - | - |  | -0.608 | 3.980 | -4.275 |
|  | *p* | - | - | - |  | 0.547 | **< .001***** | **< .001***** |
| 11.5 | *t* | - | - | - |  | -0.315 | -3.523 | -4.838 |
|  | *p* | - | - | - |  | 0.754 | **.001***** | **< .001***** |
| 12 | *t* | - | - | - |  | -0.394 | -3.127 | -5.052 |
|  | *p* | - | - | - |  | 0.696 | **< .01**** | **< .001***** |
| 12.5 | *t* | - | - | - |  | 0.070 | -3.352 | -5.195 |
|  | *p* | - | - | - |  | 0.944 | **< .01**** | **< .001***** |
| 13 | *t* | - | - | - |  | 0.507 | -3.543 | -5.046 |
|  | *p* | - | - | - |  | 0.616 | **.001***** | **< .001***** |
| 13.5 | *t* | - | - | - |  | 0.058 | -3.559 | -4.963 |
|  | *p* | - | - | - |  | 0.954 | **.001***** | **< .001***** |
| 14 | *t* | - | - | - |  | -0.318 | -3.407 | -5.413 |
|  | *p* | - | - | - |  | 0.752 | **< .01**** | **< .001***** |
| 14.5 | *t* | - | - | - |  | 0.114 | -2.316 | -6.221 |
|  | *p* | - | - | - |  | 0.910 | **< .05*** | **< .001***** |

######

##### Table S2. Pupil diameter

|  |  | Post-cue window | | |  | Post-event window | | |
| --- | --- | --- | --- | --- | --- | --- | --- | --- |
| Time (s) | Statistics | False alarm  vs. Control | Predictable  vs. Control | Unpredictable  vs. Control |  | False alarm  vs. Control | Predictable  vs. Control | Unpredictable  vs. Control |
| 0 | *t* | -1.943 | -1.765 | - |  | 1.395 | 0.804 | 0.040 |
|  | *p* | 0.060 | 0.086 | - |  | 0.172 | 0.427 | 0.968 |
| 0.5 | *t* | -0.864 | -0.459 | - |  | 1.135 | 1.046 | 1.030 |
|  | *p* | 0.393 | 0.649 | - |  | 0.264 | 0.303 | 0.310 |
| 1 | *t* | 2.178 | 2.084 | - |  | 0.276 | 0.939 | -0.076 |
|  | *p* | **< .05*** | **< .05*** | - |  | 0.784 | 0.354 | 0.940 |
| 1.5 | *t* | 4.177 | 4.161 | - |  | 0.059 | -0.500 | -0.659 |
|  | *p* | **< .001***** | **< .001***** | - |  | 0.953 | 0.620 | 0.514 |
| 2 | *t* | 5.769 | 5.205 | - |  | 1.431 | 0.331 | -1.680 |
|  | *p* | **< .001***** | **< .001***** | - |  | 0.161 | 0.743 | 0.102 |
| 2.5 | *t* | 5.955 | 5.629 | - |  | 1.339 | 0.651 | -1.725 |
|  | *p* | **< .001***** | **< .001***** | - |  | 0.189 | 0.519 | 0.093 |
| 3 | *t* | 5.822 | 4.933 | - |  | 2.271 | 0.571 | -2.786 |
|  | *p* | **< .001***** | **< .001***** | - |  | **< .05*** | 0.572 | **< .01**** |
| 3.5 | *t* | 6.544 | 5.269 | - |  | 2.725 | 0.276 | -4.302 |
|  | *p* | **< .001***** | **< .001***** | - |  | **< .05*** | 0.784 | **< .001***** |
| 4 | *t* | 6.613 | 6.143 | - |  | 2.064 | -0.193 | -6.114 |
|  | *p* | **< .001***** | **< .001***** | - |  | **< .05*** | 0.848 | **< .001***** |
| 4.5 | *t* | 7.595 | 6.674 | - |  | 2.300 | -0.437 | -8.602 |
|  | *p* | **< .001***** | **< .001***** | - |  | **< .05*** | 0.665 | **< .001***** |
| 5 | *t* | - | - | - |  | 2.699 | -0.273 | -11.450 |
|  | *p* | - | - | - |  | **< .01**** | 0.787 | **< .001***** |
| 5.5 | *t* | - | - | - |  | 4.101 | 0.054 | -13.790 |
|  | *p* | - | - | - |  | **< .001***** | 0.957 | **< .001***** |
| 6 | *t* | - | - | - |  | 4.900 | 0.151 | -15.541 |
|  | *p* | - | - | - |  | **< .001***** | 0.881 | **< .001***** |
| 6.5 | *t* | - | - | - |  | 4.872 | 0.530 | -15.610 |
|  | *p* | - | - | - |  | **< .001***** | 0.600 | **< .001***** |
| 7 | *t* | - | - | - |  | 5.160 | 0.625 | -15.793 |
|  | *p* | - | - | - |  | **< .001***** | 0.536 | **< .001***** |
| 7.5 | *t* | - | - | - |  | 5.160 | 0.254 | -13.234 |
|  | *p* | - | - | - |  | **< .001***** | 0.801 | **< .001***** |
| 8 | *t* | - | - | - |  | 5.273 | 0.561 | -11.520 |
|  | *p* | - | - | - |  | **< .001***** | 0.579 | **< .001***** |
| 8.5 | *t* | - | - | - |  | 5.185 | 0.780 | -11.316 |
|  | *p* | - | - | - |  | **< .001***** | 0.440 | **< .001***** |
| 9 | *t* | - | - | - |  | 5.700 | 0.865 | -10.109 |
|  | *p* | - | - | - |  | **< .001***** | 0.393 | **< .001***** |
| 9.5 | *t* | - | - | - |  | 4.507 | 0.972 | -9.752 |
|  | *p* | - | - | - |  | **< .001***** | 0.338 | **< .001***** |
| 10 | *t* | - | - | - |  | 4.154 | 0.877 | -8.954 |
|  | *p* | - | - | - |  | **< .001***** | 0.387 | **< .001***** |
| 10.5 | *t* | - | - | - |  | 3.766 | 1.371 | -9.060 |
|  | *p* | - | - | - |  | **< .001***** | 0.179 | **< .001***** |
| 11 | *t* | - | - | - |  | 4.422 | 1.043 | -8.126 |
|  | *p* | - | - | - |  | **< .001***** | 0.304 | **< .001***** |
| 11.5 | *t* | - | - | - |  | 4.827 | 1.029 | -7.571 |
|  | *p* | - | - | - |  | **< .001***** | 0.311 | **< .001***** |
| 12 | *t* | - | - | - |  | 4.987 | 1.073 | -7.850 |
|  | *p* | - | - | - |  | **< .001***** | 0.291 | **< .001***** |
| 12.5 | *t* | - | - | - |  | 4.375 | 0.869 | -7.621 |
|  | *p* | - | - | - |  | **< .001***** | 0.391 | **< .001***** |
| 13 | *t* | - | - | - |  | 4.638 | 1.727 | -6.380 |
|  | *p* | - | - | - |  | **< .001***** | 0.093 | **< .001***** |
| 13.5 | *t* | - | - | - |  | 4.855 | 1.351 | -6.758 |
|  | *p* | - | - | - |  | **< .001***** | 0.185 | **< .001***** |
| 14 | *t* | - | - | - |  | 4.790 | 1.441 | -5.987 |
|  | *p* | - | - | - |  | **< .001***** | 0.158 | **< .001***** |
| 14.5 | *t* | - | - | - |  | 4.419 | 1.411 | -6.192 |
|  | *p* | - | - | - |  | **< .001***** | 0.167 | **< .001***** |

#####

##### Table S3. Driving speed

|  |  | Post-cue window | | |  | Post-event window | | |
| --- | --- | --- | --- | --- | --- | --- | --- | --- |
| Time (s) | Statistics | False alarm  vs. Control | Predictable  vs. Control | Unpredictable  vs. Control |  | False alarm  vs. Control | Predictable  vs. Control | Unpredictable  vs. Control |
| 0 | *t* | 1.232 | 1.649 | - |  | 2.486 | 3.793 | 0.180 |
|  | *p* | 0.226 | 0.108 | - |  | **< .05*** | **< .001***** | 0.858 |
| 0.5 | *t* | 1.272 | 2.090 | - |  | 2.788 | 4.238 | 0.877 |
|  | *p* | -0.012 | 0.06 | - |  | **< .01**** | **< .001***** | 0.386 |
| 1 | *t* | -0.139 | 0.670 | - |  | 3.271 | 3.856 | 0.747 |
|  | *p* | 0.890 | 0.093 | - |  | **< .01**** | **< .001***** | 0.460 |
| 1.5 | *t* | -2.335 | -1.725 | - |  | 4.337 | 4.206 | 0.856 |
|  | *p* | **< .05*** | **< .01**** | - |  | **< .001***** | **< .001***** | 0.398 |
| 2 | *t* | -4.069 | -3.435 | - |  | 5.916 | 4.420 | 1.189 |
|  | *p* | **< .001***** | **< .001***** | - |  | **< .001***** | **< .001***** | 0.242 |
| 2.5 | *t* | -5.733 | -4.670 | - |  | 6.572 | 4.578 | 1.624 |
|  | *p* | **< .001***** | **< .001***** | - |  | **< .001***** | **< .001***** | 0.113 |
| 3 | *t* | -6.807 | -5.193 | - |  | 6.305 | 4.919 | 2.493 |
|  | *p* | **< .001***** | **< .001***** | - |  | **< .001***** | **< .001***** | **< .05*** |
| 3.5 | *t* | -7.001 | -5.367 | - |  | 6.081 | 5.244 | 3.834 |
|  | *p* | **< .001***** | **< .001***** | - |  | **< .001***** | **< .001***** | **< .001***** |
| 4 | *t* | -7.855 | -5.738 | - |  | 5.875 | 5.362 | 5.331 |
|  | *p* | **< .001***** | **< .001***** | - |  | **< .001***** | **< .001***** | **< .001***** |
| 4.5 | *t* | -8.939 | -6.433 | - |  | 5.906 | 5.294 | 6.826 |
|  | *p* | **< .001***** | **< .001***** | - |  | **< .001***** | **< .001***** | **< .001***** |
| 5 | *t* | - | - | - |  | 6.004 | 5.002 | 8.620 |
|  | *p* | - | - | - |  | **< .001***** | **< .001***** | **< .001***** |
| 5.5 | *t* | - | - | - |  | 6.463 | 5.187 | 9.683 |
|  | *p* | - | - | - |  | **< .001***** | **< .001***** | **< .001***** |
| 6 | *t* | - | - | - |  | 6.737 | 5.049 | 10.578 |
|  | *p* | - | - | - |  | **< .001***** | **< .001***** | **< .001***** |
| 6.5 | *t* | - | - | - |  | 7.098 | 4.581 | 11.440 |
|  | *p* | - | - | - |  | **< .001***** | **< .001***** | **< .001***** |
| 7 | *t* | - | - | - |  | 6.912 | 4.081 | 12.223 |
|  | *p* | - | - | - |  | **< .001***** | **< .001***** | **< .001***** |
| 7.5 | *t* | - | - | - |  | 6.379 | 3.723 | 12.515 |
|  | *p* | - | - | - |  | **< .001***** | **< .001***** | **< .001***** |
| 8 | *t* | - | - | - |  | 6.332 | 3.438 | 12.534 |
|  | *p* | - | - | - |  | **< .001***** | **< .01**** | **< .001***** |
| 8.5 | *t* | - | - | - |  | 6.488 | 2.931 | 11.955 |
|  | *p* | - | - | - |  | **< .001***** | **< .01**** | **< .001***** |
| 9 | *t* | - | - | - |  | 6.759 | 2.034 | 11.229 |
|  | *p* | - | - | - |  | **< .001***** | **.05*** | **< .001***** |
| 9.5 | *t* | - | - | - |  | 7.086 | 2.035 | 10.890 |
|  | *p* | - | - | - |  | **< .001***** | **< .05*** | **< .001***** |
| 10 | *t* | - | - | - |  | 6.512 | 1.558 | 10.288 |
|  | *p* | - | - | - |  | **< .001***** | 0.128 | **< .001***** |
| 10.5 | *t* | - | - | - |  | 5.137 | 1.070 | 10.368 |
|  | *p* | - | - | - |  | **< .001***** | 0.292 | **< .001***** |
| 11 | *t* | - | - | - |  | 3.400 | 0.601 | 9.889 |
|  | *p* | - | - | - |  | **< .01**** | 0.552 | **< .001***** |
| 11.5 | *t* | - | - | - |  | 1.757 | 0.098 | 9.367 |
|  | *p* | - | - | - |  | 0.088 | 0.922 | **< .001***** |
| 12 | *t* | - | - | - |  | 0.392 | -0.725 | 8.731 |
|  | *p* | - | - | - |  | 0.697 | 0.785 | **< .001***** |
| 12.5 | *t* | - | - | - |  | -0.627 | -0.618 | 8.214 |
|  | *p* | - | - | - |  | 0.535 | 0.541 | **< .001***** |
| 13 | *t* | - | - | - |  | -1.540 | -1.051 | 7.852 |
|  | *p* | - | - | - |  | 0.132 | 0.301 | **< .001***** |
| 13.5 | *t* | - | - | - |  | -2.251 | -1.405 | 7.517 |
|  | *p* | - | - | - |  | **< .05*** | 0.169 | **< .001***** |
| 14 | *t* | - | - | - |  | -2.993 | -1.611 | 7.245 |
|  | *p* | - | - | - |  | **< .01**** | 0.116 | **< .001***** |
| 14.5 | *t* | - | - | - |  | -3.706 | -2.012 | 6.783 |
|  | *p* | - | - | - |  | **< .001***** | 0.052 | **< .001***** |

##### Table S4. Raw means for the dependent variables, depending on condition type and driving experience level (standard deviations are presented in brackets).

|  | False alarm | |  | Predictable | |  | Unpredictable | |
| --- | --- | --- | --- | --- | --- | --- | --- | --- |
|  | Higher experience | Lower experience |  | Higher experience | Lower experience |  | Higher experience | Lower experience |
| Peak heart rate | 5.54  (4.13) | 7.06  (6.66) |  | 6.43  (6.05) | 9.79  (5.87) |  | 9.31  (7.25) | 13.08  (8.64) |
| Reported stress | -0,45  (0,96) | 0,14  (0,69) |  | -0,22  (0,72) | 0,25  (0,69) |  | 0,28  (1,04) | 0,82  (0,79) |
| Reported valence | -0,10  (0,77) | -0,37  (0,67) |  | -0,05  (0,85) | -0,27  (0,82) |  | -0,85  (0,68) | -0,97  (0,76) |
| Reported arousal | 0,74  (0,49) | 0,48  (0,67) |  | 0,83  (0,37) | 0,57  (0,44) |  | 0,38  (0,79) | 1,08  (0,44) |
